# Leukemic Non-Nodal Mantle Cell Lymphoma Presenting with Traumatic Splenic Rupture

**DOI:** 10.3390/hematolrep18030032

**Published:** 2026-05-13

**Authors:** Moinul Haque, Razie Amraei, Krasimira A. Rozenova

**Affiliations:** Department of Pathology, Yale University School of Medicine, New Haven, CT 06510, USA; moinul.haque@yale.edu (M.H.); razie.amraei@yale.edu (R.A.)

**Keywords:** non-nodal mantle cell lymphoma, spleen, cyclin D1

## Abstract

**Background:** Leukemic non-nodal variant mantle cell lymphoma (nnMCL) is an uncommon subtype of mantle cell lymphoma that lacks lymphadenopathy and generally follows an indolent clinical course. Adverse genetic alterations such as *TP53* inactivation and del(13q) may have prognostic significance. Clinical findings such as splenomegaly may serve as a clue to the diagnosis and should prompt further evaluation. **Case Presentation:** We describe a 91-year-old woman who presented with a one-month history of anemia (hemoglobin 12.3 g/dL), mild thrombocytopenia (platelets 136 × 10^9^/L), isolated splenomegaly and no palpable lymphadenopathy. Despite a normal total white blood cell count, intermittent relative lymphocytosis with atypical lymphocytes (4%) and smudge cells was detected on the complete blood count. Peripheral blood flow cytometry demonstrated a monoclonal kappa-restricted B-cell population negative for CD5 and CD10, comprising approximately 20% of lymphocytes. Conventional karyotyping and fluorescent in situ hybridization (FISH) analysis identified del(13q), del(17p)/*TP53*, and *IGH-CCND1* rearrangement in 8–19.5% of peripheral blood leukocytes. A month after the initial assessment, the patient presented following a fall. CT imaging of the abdomen revealed marked splenomegaly, a large subcapsular/perisplenic hematoma, and moderate-to-large hemoperitoneum. Emergent laparotomy showed an enlarged spleen (1490 g, 23 × 16 × 7.5 cm) with laceration. Histologic evaluation showed atypical lymphoid cells positive for CD20 and cyclin D1, with strong p53 expression, negative for CD5 and SOX11, and a low Ki-67 index. Similar involvement was identified in the small bowel and appendix. Targeted sequencing of splenic tissue, performed as part of a retrospective molecular characterization, identified a pathogenic *TP53* variant *(*p.His179Gln). **Conclusions:** This case provides a rare opportunity to evaluate splenic and small intestinal involvement by nnMCL at both the gross and histologic levels. It highlights the importance of integrating clinical findings with flow cytometry, imaging, cytogenetic, and molecular data in establishing the diagnosis. Even when peripheral blood findings suggest a low disease burden, imaging may better define the extent of disease and support appropriate clinical assessment, particularly in elderly patients at risk for complications related to splenomegaly.

## 1. Introduction

Mantle cell lymphoma (MCL) is a mature B-cell lymphoma that typically affects older adults, and accounts for approximately 5–7% of all non-Hodgkin lymphomas (NHLs) [1,2]. Its defining genetic hallmark is the reciprocal translocation t(11;14)(q13;q32), which involves *IGH* on chromosome 14 and *CCND1* on chromosome 11. The aberrancy leads to cyclin D1 overexpression [3], which is detectable by immunohistochemistry (IHC), and serves as a reliable surrogate marker in routine diagnostic practice. In rare cases where the t(11;14) translocation is lacking, rearrangement of *CCND2* or *CCND3* may be found [4]. Major clinicopathologic forms of MCL include classical/conventional MCL (cMCL) and the leukemic non-nodal variant (nnMCL), which differs significantly in clinical behavior and prognosis. cMCL, the more common form of MCL, has an aggressive clinical course, with frequent involvement of lymph nodes, bone marrow, and gastrointestinal tract [2]. In contrast, nnMCL, which accounts for 10–20% of MCL cases, generally follows a more indolent clinical course [5]. nnMCL often presents asymptomatically with low-level blood and bone marrow involvement and, as denoted by its name, without lymphadenopathy [6,7,8,9]. The absence of lymphadenopathy can make timely recognition of nnMCL challenging, as most mature B-cell lymphomas are diagnosed through biopsy of an enlarged lymph node. Splenomegaly is seen in approximately 42% of cases [10,11] and, when present, may serve as a clinical clue to this diagnosis. However, because splenic biopsies are rarely performed in routine practice, opportunities to assess splenic involvement histologically are limited.

Although cMCL and nnMCL share the hallmark t(11;14) rearrangement, they differ in clinical behavior, cell of origin, immunophenotype, and molecular features. cMCL is believed to arise from pre-germinal B cells located in the inner mantle zones of lymphoid follicles, whereas nnMCL is proposed to originate from post-germinal center memory B cells [3]. Supporting this distinction, nnMCL shows somatic hypermutation of the immunoglobulin heavy chain variable region (*IGHV*) genes in 90–95% of cases, compared with only 5–10% in cMCL [10].

Immunophenotypically, nnMCL differs significantly from cMCL. CD5 expression, expected in cMCL (~85–90% of cases), is reported in only 25–50% of nnMCL cases [6]. SOX11, detected in up to 95% of cMCL and helpful in the diagnosis of cyclin D1-negative cases [7], is characteristically absent in nnMCL [6,8]. Additionally, features more frequently seen in nnMCL include CD23 expression, kappa light chain restriction (up to 80% of cases) [11], and CD200 expression [9], a marker more typical of chronic lymphocytic leukemia/small lymphocytic lymphoma (CLL/SLL). This immunophenotypic overlap, together with morphologic similarities to CLL/SLL, can make nnMCL diagnostically challenging unless cyclin D1 staining and *CCND1* rearrangement are specifically evaluated [10].

Comparative next-generation sequencing studies have shown that mutations in *CDK4*, *CDKN2A*, *RB1*, *CARD11*, *SPEN*, *FBXW7* and *ATM* are more common in cMCL, whereas mutations in *NOTCH1* and *BIRC3* are more frequently observed in nnMCL [12,13]. However, considerable genetic heterogeneity exists within nnMCL. While most cases of nnMCL follow an indolent course, a subset with alterations such as 17p (*TP53*), 11q (*ATM*), or 13q14 deletions can exhibit aggressive clinical behavior, including accelerated progression, resistance to standard therapy, and poor outcomes [6]. *TP53* alterations, in particular, have been consistently associated with adverse prognosis in nnMCL across multiple studies.

Here, we report a case of nnMCL presenting with marked splenomegaly and characteristic immunophenotypic and cytogenetic findings. Traumatic rupture of the spleen led to splenectomy and partial small bowel resection, providing a rare opportunity to assess the histologic pattern of splenic and gastrointestinal involvement in nnMCL.

## 2. Case Presentation

A 91-year-old female with a medical history significant for type 2 diabetes mellitus, chronic kidney disease (stage 3), hypothyroidism, atrial fibrillation, osteoporosis, and prior colon cancer status post-resection, and no prior diagnosis of hematolymphoid malignancy, presented for routine follow-up for osteopenia-related back pain. She denied B symptoms, including fever, night sweats, and unintentional weight loss. On physical examination, splenomegaly was noted, with no palpable lymphadenopathy. Laboratory workup was notable for a one-month history of anemia (hemoglobin 12.3 g/dL), mild thrombocytopenia (platelets 136 × 10^9^/L), and intermittent relative lymphocytosis with detection of atypical lymphocytes (4%) and smudge cells, despite a normal total white blood cell count (range 6.2–7.4 K/µL). Lactate dehydrogenase (LDH) was within the normal range (208 U/L). These findings raised concern for an underlying hematologic neoplasm, prompting additional testing.

Peripheral blood flow cytometry showed involvement by a monoclonal kappa-restricted B-cell population with a non-specific CD5−/CD10− immunophenotype, comprising approximately 20% of lymphocytes. Conventional karyotype and fluorescence in situ hybridization (FISH) analysis revealed deletions in 13q (*RB1*) and 17p (*TP53*) and an *IGH-CCND1* gene rearrangement in 8–19.5% of peripheral blood leukocytes. These findings confirmed the suspicion of B-cell lymphoproliferative disease, and the patient was referred for further evaluation.

**Figure 1:**
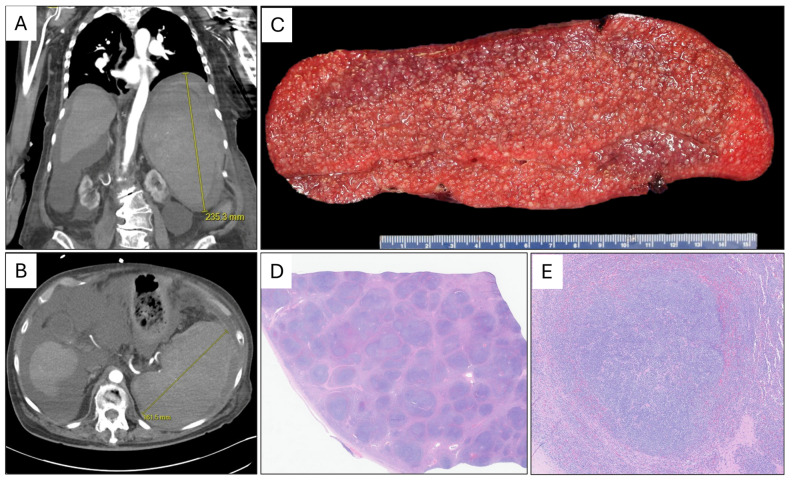
nnMCL presenting with marked splenomegaly. Coronal (**A**) and axial (**B**) CT scan views of the spleen. Gross (**C**) and representative low (**D**) and intermediate (**E**) magnification microscopic images of the spleen involved by leukemic non-nodal mantle cell lymphoma.

Approximately one month after the initial hematology assessment, the patient presented to the emergency department following a ground-level fall. On clinical examination, she was noted to be in hypotensive shock. A bedside-focused assessment with sonography (FAST) showed free fluid in the abdomen. A subsequent computed tomography (CT) scan revealed marked splenomegaly, moderate-to-large hemoperitoneum secondary to splenic injury, and a large subcapsular/peri-splenic hematoma (Figure 1A,B). Of note, a CT scan performed four years prior had demonstrated a normal spleen. An emergency laparotomy was performed, including splenectomy, partial small bowel resection with anastomosis, and appendectomy. The latter procedures were necessary due to intraoperative findings. Postoperatively, the patient’s recovery was complicated by worsening renal function and decompensation with a gradual decline in vital signs. After discussion with family, the patient was transitioned to comfort measures and died shortly thereafter.

Gross examination of the spleen showed marked enlargement (1490 g, 23 × 16 × 7.5 cm) with a faintly nodular capsule and a large surface defect (up to 17 cm), consistent with traumatic rupture. The cut surfaces were rubbery and red with prominent expansion of the white pulp (Figure 1C).

**Figure 2:**
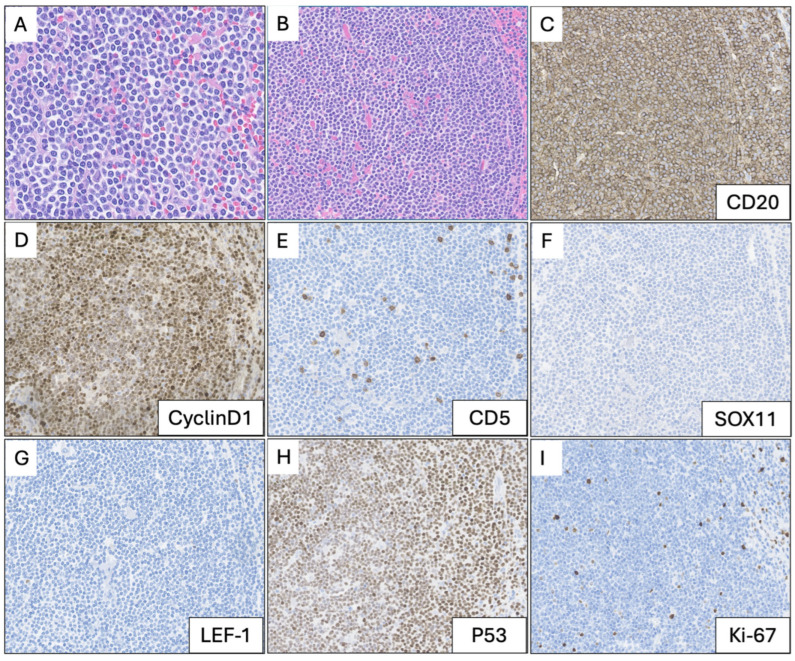
High-power (80×, (**A**); 40×, (**B**)) photomicrographs of leukemic non-nodal mantle cell lymphoma with corresponding immunohistochemical stains: CD20 (**C**), cyclin D1 (**D**), CD5 (**E**), SOX11 (**F**), LEF-1 (**G**), p53 (**H**), and Ki-67 (**I**).

Histologic sections of the spleen parenchyma showed expanded white pulp (Figure 1D,E). The infiltrate was composed of small- to intermediate-sized atypical lymphoid cells with mildly irregular nuclear contours, clumped chromatin, inconspicuous nucleoli, and scant cytoplasm (Figure 2A,B).

**Figure 3:**
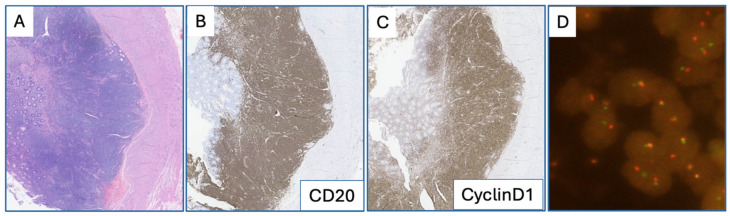
Gastrointestinal involvement of leukemic non-nodal mantle cell lymphoma. H&E section showing lymphoid infiltrate (**A**). Immunohistochemistry showing CD20-positive B cells co-expressing cyclin D1 (**B**,**C**). FISH demonstrating *CCND1* rearrangement (**D**).

Immunohistochemical (IHC) stains showed that the neoplastic cells were positive for CD20, cyclin D1, and strong p53 expression and were negative for CD5, SOX11, and LEF-1. The Ki-67 proliferation index was low (<10% of cells) (Figure 2I). The appendix and small bowel were grossly unremarkable but histopathologic examination showed expansion of the lamina propria and subserosal nodular infiltrates of cyclin D1-positive atypical lymphoid cells (Figure 3). The appendix also showed mucosal and submucosal involvement. FISH with a *CCND1* break-apart probe confirmed the presence of a *CCND1* rearrangement (Figure 3D), consistent with findings in the peripheral blood, where an *IGH-CCND1* rearrangement was identified (using a dual fusion FISH probe). Targeted next-generation sequencing of involved splenic tissue, retrospectively performed for molecular characterization, identified a pathogenic *TP53* variant (NM_000546.6(*TP53*):c.537T>G (p.His179Gln)) with a variant allele fraction (VAF) of 57%, supporting loss of heterozygosity at the *TP53* locus in the context of the deletion of 17p13 identified by cytogenetic studies.

The full immunophenotype of the presented case by flow cytometry and IHC is summarized in Table 1.

## 3. Discussion

In current practice, nnMCL is typically diagnosed based on peripheral blood and bone marrow findings, and splenic tissue is infrequently available for direct evaluation. In this case, a patient with newly identified nnMCL suffered splenic rupture after a fall, and the trauma-related splenectomy provided a rare opportunity to evaluate splenic involvement at both the gross and histologic levels. The spleen showed prominent expansion of the white pulp by small atypical lymphoid cells, with minimal red pulp involvement, in keeping with proposed patterns of disease distribution. Prior reports have described splenic involvement by nnMCL with similar histomorphologic features, although detailed gross–histologic correlation has been limited. Fang et al. described splenic involvement by nnMCL with mainly white pulp involvement, while Gorodetskiy et al. reported a more subtle pattern with cyclin D1-positive, SOX11-negative lymphocytes localized to the inner mantle zones of splenic follicles and only small clusters in the red pulp. Orchard et al. further highlighted the importance of tissue histology, including the spleen when available, in resolving diagnostic uncertainty in non-nodal t(11;14)-positive lymphocytosis [14,15,16]. Together, these reports support the concept that splenic involvement in nnMCL may span a spectrum from subtle mantle zone-restricted disease to more extensive white pulp expansion, as seen in the present case.

Splenomegaly is observed in approximately 42% of nnMCL cases [10]. While it is recognized as a clinical feature, its relationship to disease biology and prognosis remains unclear. Current risk stratification models rely primarily on clinical and molecular parameters rather than the extent of splenic enlargement. Nevertheless, marked splenomegaly increases the risk of splenic rupture, particularly in the setting of trauma, which constitutes a medical emergency.

In this case, nnMCL was diagnosed shortly before the patient’s death, and therapy was not initiated because the patient’s condition deteriorated following traumatic splenic rupture. A CT scan obtained four years earlier showed a normal spleen, and the disease course in the intervening period cannot be determined. The absence of prior hematologic workup precludes definitive conclusions regarding the timeline or tempo of disease progression. However, *TP53* inactivation identified in the neoplastic cells raises the possibility that this may not have represented a truly indolent phase, but rather an evolving disease with early molecular changes associated with more aggressive biologic behavior.

The integration of data from clinical, imaging, genetic, and histopathologic assessment is important for non-Hodgkin B-cell lymphomas, such as nnMCL. For example, flow cytometric analysis and complete blood count alone showing monotypic, kappa-restricted B cells may only suggest low-level involvement by B-cell lymphoproliferative disease, best classified as monoclonal B-cell lymphocytosis based on absolute lymphocyte counts, unless splenomegaly is identified clinically or radiographically. A particularly difficult histopathologic differential diagnosis of nnMCL is CLL/SLL, due to overlapping findings such as morphologic features of the neoplastic cells, clonal B cells expressing CD200 by flow cytometry, occasional CD23 expression by IHC, and del(13q). Of note, some authors have suggested that there is significant biological similarity between atypical CLL/SLL and nnMCL [9]. Rarely, CLL/SLL can acquire *IGH-CCND1* as a secondary genetic abnormality, as well as del(17p) [17,18]. In this case the clinical presentation, low Ki-67 proliferation index, lack of CD5, CD23, and LEF-1 expression argued against CLL/SLL. Without cyclin D1 immunohistochemistry or cytogenetic confirmation of *CCND1* rearrangement, nnMCL can sometimes be misdiagnosed. This highlights the importance of performing a complete diagnostic workup, particularly when evaluating splenomegaly or unusual small B-cell populations. Table 2 summarizes the differential diagnoses for the current case, highlighting the morphologic and immunophenotypic features of other B-cell lymphomas commonly involving the spleen and noting key diagnostic distinctions between entities. More detailed reviews of B-cell lymphomas involving the spleen are available elsewhere [19,20].

Timely and accurate diagnosis of nnMCL is essential because, although an initial “watch-and-wait” approach may be appropriate for many patients, the disease can progress or transform into aggressive forms, including blastoid or pleomorphic variants, which require treatment [21]. The relationship between nnMCL and cMCL remains an area of ongoing discussion. Although both share the hallmark t(11;14)(q13;q32) translocation, they differ significantly in clinical behavior, immunophenotype, and molecular landscape. *TP53* alterations have been consistently associated with adverse prognosis in mantle cell lymphoma, including the nnMCL variant. Although additional cytogenetic or molecular abnormalities, such as ATM aberrations, deletion 13q14, or a complex karyotype, may further contribute to aggressive clinical behavior and an earlier need for therapy [6,13,21,22,23], *TP53* inactivation itself is recognized as an important adverse feature, even in the absence of high-grade morphology or an elevated proliferation index. In the patient presented here, several clinically relevant findings, including imaging studies, low Ki-67 proliferation index, strong p53 expression by IHC, and the *TP53* pathogenic variant (identified retrospectively), became fully apparent only after the traumatic event and subsequent evaluation of the surgically resected spleen. In conjunction with cytogenetic abnormalities, including del(13q) and del(17p) detected in peripheral blood, as well as a higher MIPI score driven in part by advanced age, these additional findings may have influenced clinical assessment and prompted consideration of therapy.

## 4. Conclusions

When a clonal CD5−/CD10− kappa-restricted B-cell population is identified by flow cytometry and/or immunohistochemistry, the differential diagnosis often includes chronic lymphocytic leukemia/small lymphocytic lymphoma, marginal zone lymphoma, and other splenic B-cell lymphomas in the right clinical context. However, this immunophenotype also includes nnMCL, especially in the presence of splenomegaly and with minimal or no lymphadenopathy. Immunohistochemistry for cyclin D1 and cytogenetic analysis for *CCND1* rearrangement are critical and, in most cases, sufficient for diagnosis. The recognition of nnMCL at diagnosis is essential for management and clinical follow-up. This case highlights the potential discordance between low-level peripheral blood involvement and extensive extranodal disease.

## Figures and Tables

**Table 1:** Immunophenotypic findings of the presented case of leukemic non-nodal mantle cell lymphoma by flow cytometry and immunohistochemistry.

Method	Immunophenotypic Findings
Flow Cytometry	CD20+, CD19+, CD5−, CD23−, CD10−, CD103−, CD38−, FMC7−, CD43−, kappa light chain restricted
Immunohistochemistry	Positive: CD20, cyclin D1, p53 (3+ expression seen in >50% of cells). Negative: CD3, LEF-1, CD5, SOX11. Ki-67 is low (<10% of cells)

**Table 2:** Differential diagnosis of the current case with isolated splenomegaly. Morphologic and immunophenotypic findings of B-cell lymphomas commonly involving the spleen *.

	Morphologic Findings	Immunophenotypic Findings
Leukemic non-nodal mantle cell lymphoma	White pulp expansion with red pulp infiltration; monotonous small lymphocytes with clumped chromatin	CD19+ CD20+ CD5+/− Cyclin D1+ SOX11- CD23-
Chronic lymphocytic leukemia/small lymphocytic lymphoma	White pulp nodules with coalescence of follicles; monotonous small lymphocytes with clumped chromatin	CD19+ CD20dim+ CD5+ CD23+ CD10− LEF1+ CD200+
Splenic marginal zone lymphoma	Nodular/micronodular white pulp expansion with biphasic pattern; often red pulp infiltration; small lymphocytes and marginal zone cells	CD19+ CD20+ CD5− CD10− CD23− IgM+ IgD+
Classical hairy cell leukemia	Diffuse red pulp infiltration with atrophic white pulp; small to medium-sized lymphocytes with abundant cytoplasm and central nuclei; cells with hairy projections	CD19+ CD20+ CD5− CD11c+ CD103+ CD123+ CD25+ Annexin A1+ BRAF V600E+
Hairy cell leukemia variant	Diffuse red pulp infiltration; atrophic white pulp; variable nuclear morphology of classic hairy cell leukemia and prolymphocytes	CD19+ CD20+ CD5− CD11c+ CD103+ CD123− CD25− Annexin A1− BRAF V600E−
Splenic diffuse red pulp small B-cell lymphoma	Diffuse pattern of red pulp infiltration in the sinusoids and cords; partial atrophy of white pulp; monotonous small to medium-sized lymphocytes with round nuclei and pale cytoplasm	CD19+ CD20+ CD5− CD10− CD23− CD123− CD25− Annexin A1− IgG+

* Features listed represent typical, concise findings and are not exhaustive; deviations from classic morphologic and immunophenotypic profiles may occur.

## Data Availability

The original contributions presented in this study are included in the article. Further inquiries can be directed to the corresponding authors.

## Abbreviations

The following abbreviations are used in this manuscript:

MCL	Mantle cell lymphoma
NHL	Non-Hodgkin lymphoma
cMCL	Classical/conventional MCL
nnMCL	Leukemic non-nodal variant MCL
IGHV	Immunoglobulin heavy chain variable region
CLL/SLL	Chronic lymphocytic leukemia/small lymphocytic lymphoma
IHC	Immunohistochemistry
FISH	Fluorescent in situ hybridization
